# P-1314. Assessment of the Effect of WHO and USA Antibiotic Awareness Campaigns on Search Engine Queries for Antimicrobial Resistance

**DOI:** 10.1093/ofid/ofae631.1495

**Published:** 2025-01-29

**Authors:** Melissa Whitman, Aldo Barajas-Ochoa, Laura Pedersen

**Affiliations:** Virginia Commonwealth University, Richmond, Virginia; Virginia Commonwealth University, Richmond, Virginia; Virginia Commonwealth University, Richmond, Virginia

## Abstract

**Background:**

Antimicrobial resistance (AMR) is a public health threat according to the World Health Organization (WHO), affecting all countries and at all income levels. Since the WHO’s initial action plan in 2001, multiple campaigns have arisen such as the WHO AMR Awareness Week and the CDC’s U.S. Antibiotic Awareness Week (November 18 to 24). Here, we use Google Trends (GTr) to assess the effects of three awareness campaigns on online search queries related to AMR in the US from their inception to April 2024.

**Figure:**
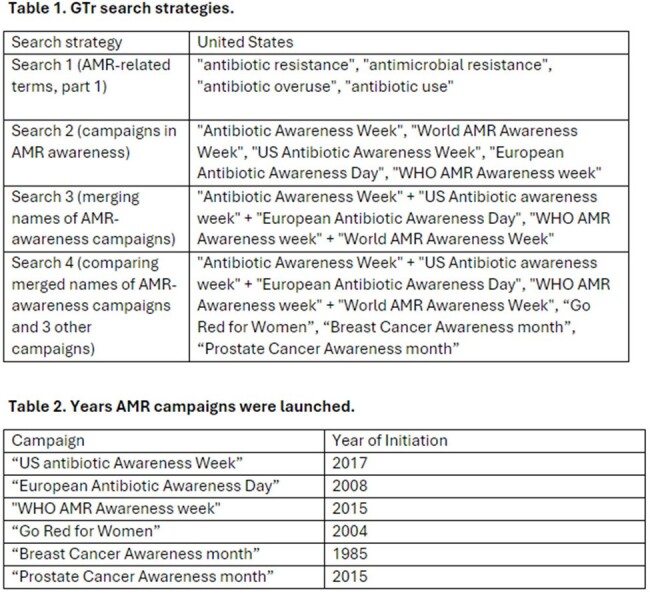

**Methods:**

GTr analyzes a sample of Google® web searches and allows users to make relative comparisons among searched terms by reporting the highest search volume as 100 and all other search volumes as a proportion of 100. Search queries related to AMR and AMR-related campaigns were used in GTr to generate normalized relative search volumes (RSVs) for each term (Table1). AMR-related campaigns' search volumes were compared to other established disease campaigns (Table 2). GTr search data from Jan 1, 2005 to April 20, 2024 was used. Descriptive statistics were used. No inferential statistics were used due to the nature of the data.

**Figure:**
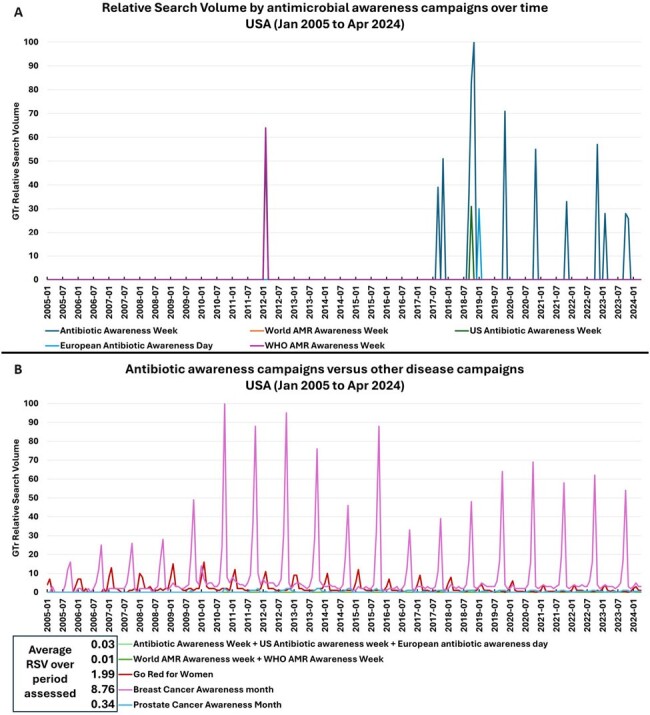

**Results:**

When assessing RSVs for AMR-related campaigns, “Antibiotic Awareness week” had the highest RSV and peaked each November after 2017 (Figure 1a). When compared to established campaigns for other diseases, the average RSVs over time for AMR-related campaigns was smaller by a ratio of 8 (prostate cancer) to 219 times (breast cancer) (Figure 1b). Amongst AMR-related terms, “antibiotic resistance” was more searched over other terms and had only a slight increase in RSV over the years (Figure 2a). Notably, RSVs for “antibiotic resistance” each year peaked in Feb-Apr and not in November, the month of the AMR campaign (Figure 2b and 2c).

**Figure:**
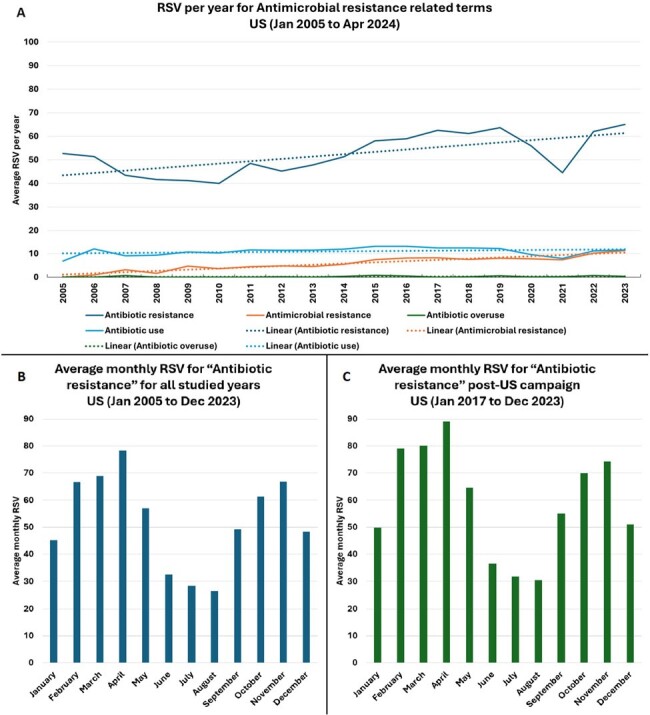

**Conclusion:**

Despite AMR being one of the most concerning public health threats in the coming decade, public knowledge of this issue trails behind other health related campaigns. RSVs related to AMR did not increase over time despite awareness efforts. Although AMR-related campaigns take place in November, it was not the month with highest RSVs. These findings suggest that more resources may be needed to support the success of these campaign efforts.

**Disclosures:**

**All Authors**: No reported disclosures

